# Diagnostic Challenges in a Hybrid Congenital Pulmonary Airway Malformation: A Case Report

**DOI:** 10.7759/cureus.104175

**Published:** 2026-02-24

**Authors:** Nelofar P, Sunita Bajaj, Sonu Rahul Tej Gaddam, Laxmi Kanth Manthri Ashok, Siri Sanmayi Medicherla, Anjali Vadakanti, Bhanu Prakash E

**Affiliations:** 1 Radiodiagnosis, Osmania Medical College, Hyderabad, IND; 2 Internal Medicine, Osmania Medical College, Hyderabad, IND

**Keywords:** aberrant systemic feeding artery, adolescent presentation of congenital lung malformation, bronchopulmonary foregut malformation spectrum, computed tomography angiography (cta), congenital pulmonary airway malformation (type 1), hybrid congenital lung lesion, intralobar pulmonary sequestration, recurrent lower respiratory tract infection

## Abstract

Congenital pulmonary airway malformation (CPAM) and bronchopulmonary sequestration are rare congenital lung anomalies that can occasionally coexist as a “hybrid” lesion. We report a case of a 15-year-old female with a two-week history of fever with evening-predominant spikes, cough, and right hypochondrial pain. The pain was aggravated by deep inspiration and coughing. Imaging revealed a right lower lobe consolidation containing air-filled cystic spaces on chest X-ray and computed tomography (CT). An anomalous arterial supply from the descending thoracic aorta on CT angiography was also noted. These findings were diagnostic of a hybrid lesion combining features of CPAM and intralobar pulmonary sequestration. This case highlights the rarity of such CPAM-sequestration hybrid lesions and the crucial role of imaging, particularly contrast-enhanced CT, in diagnosing congenital lung malformations in adolescents presenting with atypical or non-resolving pneumonia. Awareness of these rare entities is important, as timely surgical management can prevent recurrent infections and other complications.

## Introduction

Congenital lung malformations include a spectrum of developmental anomalies, of which congenital pulmonary airway malformation (CPAM) and pulmonary sequestration (PS) are among the most recognized [1]. These lesions are uncommon, with an estimated incidence of approximately 1 in 10,000-35,000 live births [2]. CPAM is characterized by cystic or adenomatous overgrowths of terminal bronchioles and abnormal alveolar structure. In contrast, PS is a dysplastic lung tissue that has no connection to the normal bronchial tree and receives systemic arterial blood supply. Pulmonary sequestration is further classified into intralobar sequestration (ILS), which lies within a normal lobe sharing the visceral pleura, and extralobar sequestration (ELS), which has its own pleural covering. About 75% of these sequestrations are intralobar [1].

Although CPAM and sequestration are distinct entities, they are now understood to sometimes coexist in the same lesion [3,4]. These composite lesions are referred to as hybrid lesions and are defined as a congenital cystic lung malformation (CPAM) with an anomalous systemic arterial supply [3,4]. Hybrid CPAM-sequestration lesions are rare [3], and many are detected on prenatal imaging or in infancy. However, some cases escape early detection and present later in childhood or even adulthood, often in the form of recurrent infection [1]. Our patient is a 15-year-old girl who presented with non-resolving pneumonia, which was ultimately diagnosed as a rare hybrid CPAM with intralobar sequestration. This case highlights the importance of recognizing congenital lung lesions in older children and the pivotal role of imaging in establishing the diagnosis.

## Case presentation

Patient information

A 15-year-old female, with no known comorbidities and unremarkable past medical history, presented with a two-week history of fever and cough. The fever was low-grade with an evening rise in temperature. She also reported a dull pain in the right hypochondrium for the same duration, which worsened on coughing and deep inspiration, suggestive of pleuritic character. There was no history of night sweats, weight loss, or chronic cough to suggest tuberculosis, and she denied any contact with TB patients. She had no prior episodes of similar respiratory illness and no known congenital anomalies. Family history was non-contributory. Prior to referral, she received oral azithromycin for 5 days at a local clinic; however, symptoms persisted with ongoing fever and cough, prompting further evaluation.

Clinical findings

On examination at presentation, the patient was febrile (temperature 38.5°C) and appeared mildly ill but was not in any acute distress. Respiratory rate was 22 breaths/minute with occasional coughing, and oxygen saturation was 98% on room air. Examination of the chest revealed reduced breath sounds and dullness to percussion over the right lower lung field. No audible crackles were noted. There was mild tenderness in the right hypochondrial region on deep inspiration, consistent with pleural irritation. Heart sounds were normal, and there were no murmurs. The remainder of the systemic examination was unremarkable, with no lymphadenopathy or hepatosplenomegaly. Initial laboratory studies showed leukocytosis of 15,000/µL (reference range: 4,000-11,000/µL) with neutrophil predominance, and C-reactive protein (CRP) was 57 mg/L (reference range: <5 mg/L), consistent with an inflammatory process. Liver and renal function tests were within normal limits.

Diagnostic assessment

**Figure 1:**
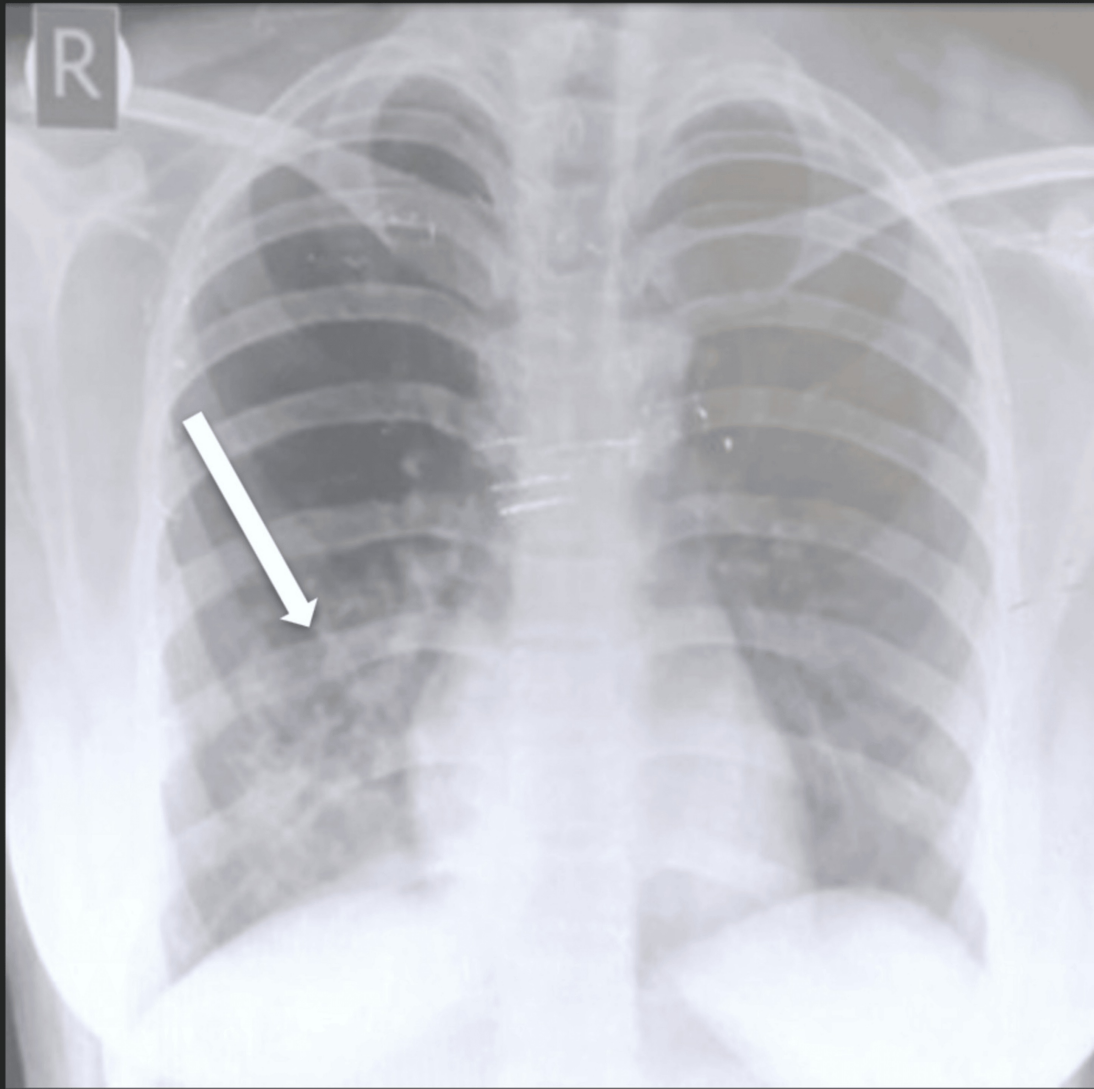
Chest radiograph (posteroanterior view) demonstrating an inhomogeneous opacity in the right lower lung zone with ill-defined margins and internal lucencies (air-filled cystic spaces).

A frontal chest X-ray showed an inhomogeneous opacity in the right lower lung zone with ill-defined borders and a few lucent (air-filled) areas within the opacity (Figure 1). This suggested a consolidation with possible cavitation or cystic components. Due to the unusual radiographic appearance and persistent symptoms, a contrast-enhanced computed tomography (CT) scan of the thorax was performed for further evaluation.

**Figure 2:**
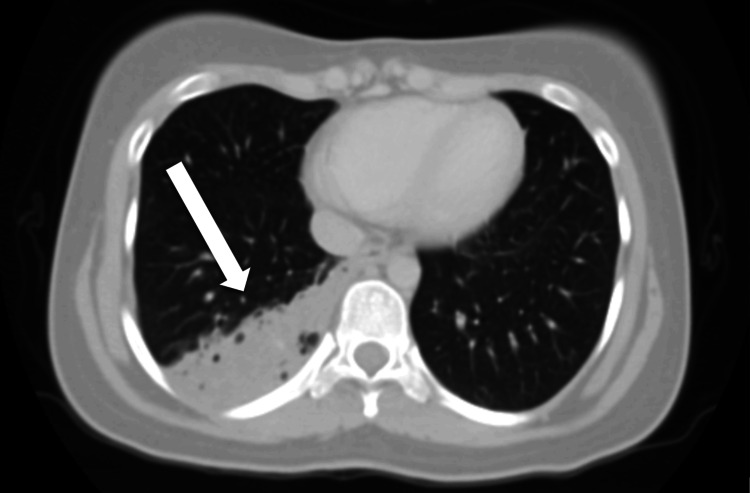
Non-contrast CT thorax (lung window) Axial image showing right lower lobe posterior segment consolidation containing air-filled cysts. CT: computed tomography

**Figure 3:**
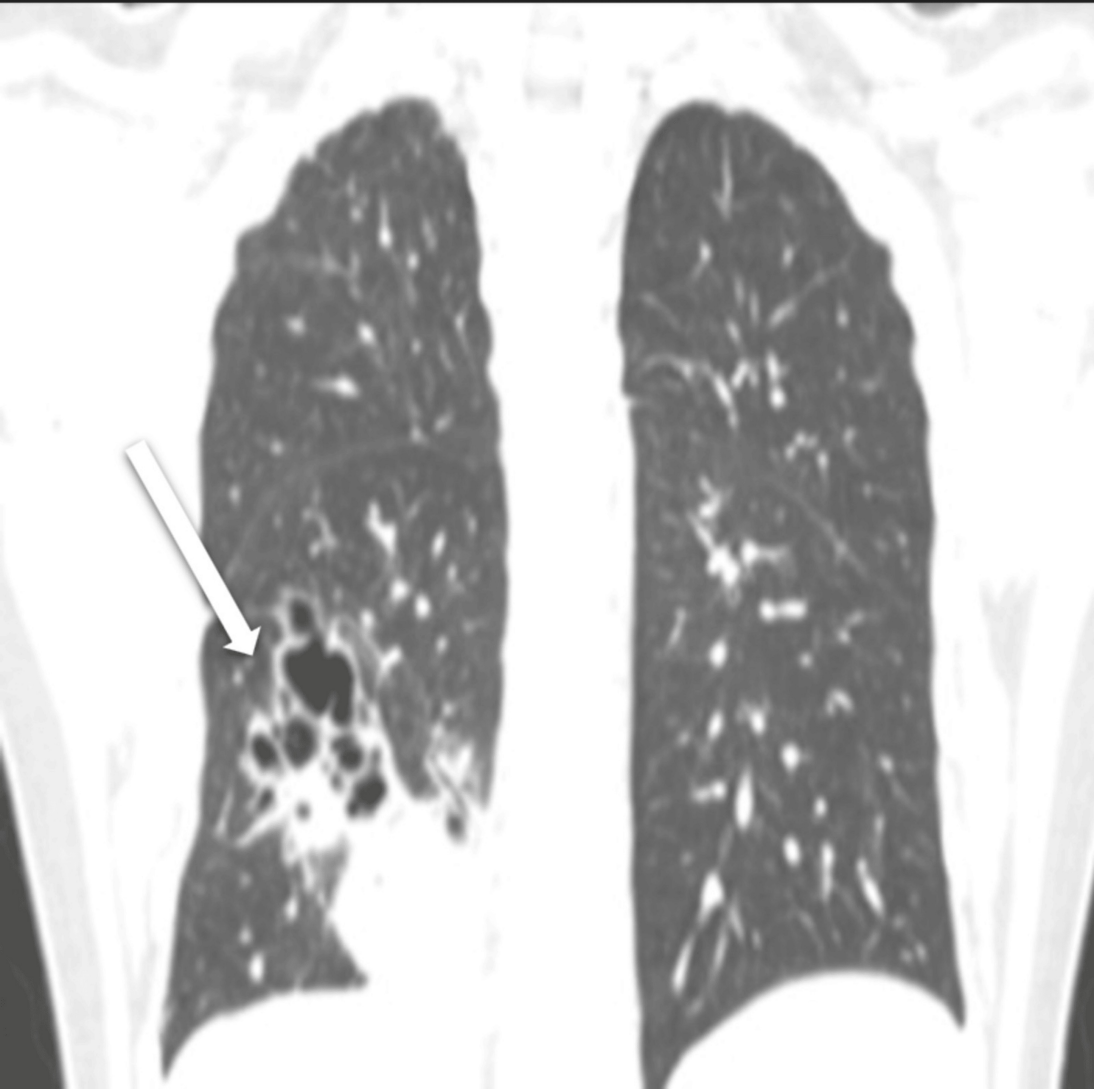
Non-contrast CT thorax (lung window) Coronal image showing right lower lobe posterior segment consolidation containing air-filled cysts. CT: computed tomography

**Figure 4:**
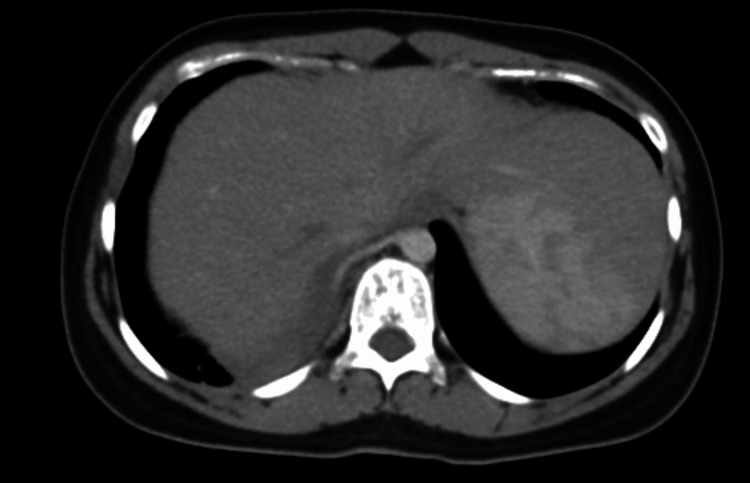
Contrast-enhanced CT angiography (mediastinal window) Axial image showing a systemic arterial feeder arising from the descending thoracic aorta supplying the right lower lobe lesion. CT: computed tomography

**Figure 5:**
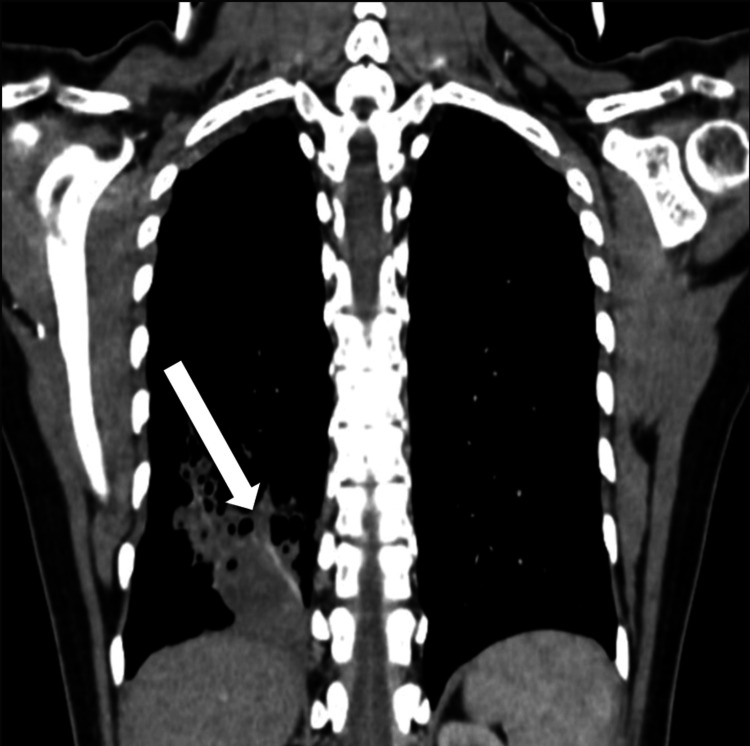
Contrast-enhanced CT angiography (mediastinal window) Coronal image showing a systemic arterial feeder arising from the descending thoracic aorta supplying the right lower lobe lesion. CT: computed tomography

The CT scan (including angiographic phases), reviewed with multiplanar reformats, including the lung window (Figures 2, 3) and soft tissue window (Figures 4, 5) to delineate the systemic feeder, revealed an area of parenchymal consolidation in the posterior segment of the right lower lobe containing multiple air-filled, thin-walled cysts with additional smaller cysts. These cystic spaces were interspersed within the consolidated lung, consistent with a cystic lung malformation (likely Type I CPAM). Notably, the CT also demonstrated an aberrant systemic arterial feeder arising from the descending thoracic aorta and supplying the consolidated cystic region of the right lower lobe. This anomalous systemic artery was seen entering the lesion (with a caliber of approximately 5 mm on CT). There were no enlarged hilar or mediastinal lymph nodes. No other focal lesions were identified in the lungs.

The combination of cystic lung changes and an independent systemic arterial supply on imaging confirmed the diagnosis of a hybrid lesion. Specifically, this was a congenital pulmonary airway malformation coexisting with an intralobar pulmonary sequestration in the right lower lobe. No invasive diagnostic procedures were pursued, as the imaging findings were considered definitive for this congenital anomaly. Microbiological work-up for infectious causes was unremarkable as sputum was negative for acid-fast bacilli on smear (making tuberculosis unlikely), and no pyogenic bacteria grew on culture, possibly due to prior antibiotic therapy. The working diagnosis was that the patient’s non-resolving pneumonia was, in fact, an infection superimposed on an underlying hybrid CPAM-sequestration malformation. Based on imaging, the patient was ultimately diagnosed as a hybrid lesion involving a Type I CPAM and intralobar pulmonary sequestration in the right lower lobe.

Therapeutic intervention

The patient received intravenous ceftriaxone (50 mg/kg/day) plus azithromycin (10 mg/kg on day 1, then 5 mg/kg/day) for 7 days. Her fever subsided by day 3, and CRP decreased from 57 mg/L to 18 mg/L (reference range: <5 mg/L). She was referred to the cardiothoracic surgery (CTVS) team for further management.

## Discussion

This case represents a rare hybrid congenital lung lesion combining CPAM and intralobar sequestration, presenting in adolescence. Most congenital pulmonary airway malformations and sequestrations are identified in neonates or infants, often via prenatal ultrasound or early postnatal imaging, especially if they cause respiratory distress at birth. However, smaller or anatomically favorable lesions may remain asymptomatic for years. In such instances, an intercurrent respiratory infection may be the first event that brings the dormant lesion to attention [2]. Our patient, at 15 years old, had no prior symptoms until an infection precipitated clinical signs of pneumonia. This suggests that a subset of patients with congenital lung anomalies may remain latent and only present when stressed by infection or other factors.

Hybrid CPAM-sequestration lesions are uncommon. In a review of pediatric cases, bronchopulmonary sequestration has been found to co-occur with CPAM (particularly CPAM type II) in some instances [1]. In fact, retrospective analyses of resected sequestrations in infants have shown a significant proportion harbor coexisting CPAM [5]. This has fueled the notion that CPAM and sequestration may not be entirely separate entities, but rather represent points on a spectrum of bronchopulmonary foregut malformations [3]. The developing lung might encounter an insult or aberration in early gestation that leads to both abnormal airway development (cyst formation) and aberrant vascular connections.

Congenital pulmonary airway malformation is most commonly classified using the modified Stocker classification [6], which is based on the anatomic level of airway maldevelopment and the gross/microscopic appearance.

**Table 1:** Modified Stocker classification (Types 0–IV) Radiology-oriented summary of the expanded (modified) Stocker classification of congenital pulmonary airway malformation (CPAM) (Types 0–IV) [6], including level of lesion, typical imaging appearance, and key clinical points. Original table created by the authors.

Stocker type	Level of lesion	Typical imaging appearance	Key points
Type 0	Tracheobronchial	Usually solid/small lungs	Incompatible with life and very rare
Type I	Bronchial/bronchiolar	Dominant large cyst, usually >2 cm; thin-walled	Best prognosis
Type II	Bronchiolar	Multiple small cysts, usually <2 cm; appears “spongy”	Often associated with other congenital anomalies
Type III	Bronchiolar/alveolar duct	Microcystic to solid-appearing mass on CT/X-ray	Can cause mass effect and mediastinal shift in infants
Type IV	Distal acinar (peripheral)	Large peripheral thin-walled cysts	

Imaging remains the cornerstone in the diagnostic workup of hybrid lesions. In our case, the chest X-ray, while abnormal, was non-specific and showed a non-resolving consolidation with some lucent areas that could have been misinterpreted as post-pneumonic changes. Subsequent CT angiography was diagnostic, definitively identifying an anomalous systemic feeder vessel. According to recent radiologic literature, CT with intravenous contrast is invaluable for characterizing congenital lung malformations. CPAM typically appears as a multicystic air-filled mass (the appearance varies by subtype), whereas sequestration is identified by an anomalous systemic artery on imaging. In our patient, the CT demonstrated features of a CPAM Type I lesion (multiple air-filled, thin-walled cysts with additional smaller cysts) alongside the hallmark aberrant artery indicative of a sequestration [7]. This dual finding confirmed the hybrid nature. In similar published cases, imaging findings were very much alike. A report of a 10-year-old with cough and hemoptysis showed a vascularized right lower lobe mass with air-filled cysts and a feeding vessel from the aorta on CT, exactly paralleling our observations [4]. It is noteworthy that advanced imaging, such as CT or magnetic resonance angiography (MRA), is often required to differentiate a hybrid lesion from other causes, as plain radiographs cannot delineate the vascular supply [4].

The presenting scenario of fever, productive cough, and a focal pulmonary consolidation with cystic changes raised several differential diagnoses. Table 2 summarises the common differentials encountered in clinical practice with similar imaging and presentation.

**Table 2:** Common differentials encountered in clinical practice with similar imaging and presentation. CT: computed tomography; MRI: magnetic resonance imaging; CPAM: congenital pulmonary airway malformation; MRA: magnetic resonance angiography

Differentials	CT appearance	Key discriminator in this case	MRI features
Pure CPAM (no sequestration)	Multicystic lesion ± infection	No systemic feeder	No systemic feeder on MRA
Necrotizing pneumonia/lung abscess	Consolidation with cavitation/air-fluid levels	Thick-walled cavities, clinical sepsis; no systemic feeder	Restricted diffusion in pus; enhancement pattern of abscess wall
Bronchiectasis / post-infectious pneumatoceles	cysts within consolidation	Multiple segments associated with airways; no systemic feeder	MRI is not typically needed

Management considerations

The definitive treatment for a hybrid CPAM-sequestration lesion is surgical resection of the affected lung segment or lobe. Pulmonary lobectomy (or segmentectomy, if anatomically feasible) is the treatment of choice for bronchopulmonary sequestration and is generally recommended even in asymptomatic patients. The rationale for surgery, even in the absence of symptoms, is to prevent future complications, such as recurrent pulmonary infections (as sequestered lung is prone to infection), hemorrhage (especially if there is systemic arterial supply that can bleed into airways), and very rarely malignant transformation in the malformative tissue. In CPAM, particularly type I lesions, there have been rare associations with malignancies such as pleuropulmonary blastoma or bronchioalveolar carcinoma, though the absolute risk is low. Nevertheless, this risk is eliminated by removing the abnormal tissue. In hybrid lesions, the risk of recurrent pneumonia is significant, as demonstrated in our case and others, and thus, resection is typically advised once the diagnosis is confirmed [1]. The optimal timing for surgery is usually after control of any acute infection. Our patient was referred to a cardiothoracic surgery center for further management.

## Conclusions

In summary, this case illustrates a rare hybrid pulmonary lesion presenting as a non-resolving pneumonia in an adolescent. It underlines the importance of considering congenital etiologies in atypical pneumonia cases and demonstrates the role of imaging in confirming the diagnosis. The case adds to the growing awareness that hybrid CPAM-sequestration malformations, while uncommon, are clinically significant and surgically curable. Early recognition and appropriate referral for surgical management can prevent prolonged morbidity and ensure a good outcome for patients.
